# Investigation of Metal Toxicity on Microalgae *Phaeodactylum tricornutum*, Hipersaline Zooplankter *Artemia salina*, and Jellyfish *Aurelia aurita*

**DOI:** 10.3390/toxics11080716

**Published:** 2023-08-20

**Authors:** Borja Mercado, Nuria Valero, Luis Roca-Pérez, Elena Bernabeu-Berni, Oscar Andreu-Sánchez

**Affiliations:** 1 Ocean Ecostructures, Inc., Rambla de Catalunya,14-7°-1ª, 08007 Barcelona, Spain; borjamercado@oceanecostructures.com; 2 Xenobiotics, S.L., Parque Científico de la Universitat de València, 46980 Paterna, Spain; 3 Departamento de Biología Vegetal, Facultad de Farmacia, Universitat de València, 46100 Burjassot, Spain; oscar.andreu@uv.es

**Keywords:** *Phaeodactylum tricornutum*, *Artemia salina*, *Aurelia aurita*, chromium, copper, cadmium

## Abstract

The escalating global anthropogenic activities associated with industrial development have led to the increased introduction of heavy metals (HMs) into marine environments through effluents. This study aimed to assess the toxicity of three HMs (Cr, Cu, and Cd) on organisms spanning different trophic levels: *Phaeodactylum tricornutum* (a primary producer), *Artemia salina* (a primary consumer), and *Aurelia aurita* (a secondary consumer). The EC_50_ values obtained revealed varying relative toxicities for the tested organisms. *Phaeodactylum tricornutum* exhibited the highest sensitivity to Cu, followed by Cd and Cr, while *Artemia salina* displayed the highest sensitivity to Cr, followed by Cu and Cd. *A. aurita*, on the other hand, demonstrated the highest sensitivity to Cu, followed by Cr and Cd. This experimental investigation further supported previous studies that have suggested *A. aurita* as a suitable model organism for ecotoxicity testing. Our experiments encompassed sublethal endpoints, such as pulsation frequency, acute effects, and mortality, highlighting different levels of sensitivity among the organisms.

## 1. Introduction

Nowadays, thousands of pollutants reach the marine environment and exert different types of stress and damage on organisms, resulting in negative changes in water quality and ecosystems [1]. Most of them are discharged in the marine environment as a result of countless anthropogenic activities affecting the environment [2,3]. These waste types can be different in nature and include, among others, heavy metals (HMs), detergents, microfibers, or (micro)plastics, which all contribute to the current aquatic pollution problems [4].

In this sense, marine ecosystems are one of the systems most affected by pollution because humans have used them as a dumping ground for their waste, disregarding their complexity and dynamics. Among the pollutants, the accumulation of HMs in marine ecosystems is of vital importance because they can have devastating effects on the ecological balance of the environment and biodiversity [5,6]. It would be beneficial to clarify that, among the metals, some are essential elements that play biological roles but can be toxic at high concentrations, while others are non-essential and do not have known biological functions. It is well-known that chronic HM exposure can have serious long-term health effects [5,7,8].

Aquatic pollution by HMs is related to high levels of Cd, Cr, Cu, Hg, Ni, Pb, and Zn, among others. Of these HMs, Cd, Cu, Hg, and Zn, jointly the metalloid As, are the five elements with the strongest potential impact because of their high toxicity and persistence in all aquatic ecosystems. High concentrations of these pollutants enter the environment through storm water and wastewater discharges as a consequence of agriculture and industrial activity [9,10]. When considering the source of these important HMs, Zn and Cu are present in fertilizers, while Cd and Hg are components of some fungicides and algaecides [11].

HMs’ ecological significance lies in their persistent presence, leading to accumulation in water reservoirs, integration into the food chain, and subsequent ecological harm [12,13]. Ecotoxicology, the science that examines the impact of substances on ecosystems, is closely linked to the vital need for monitoring pollution to effectively curb aquatic harm [14,15].

Pollution of aquatic areas by HMs, often called trace metals, can be detected by water and sediment analyses or by employing bioindicator organisms [16]. Some authors [17] believe that responses of biological origin can be considered more representative than the data provided by chemical or physical detectors because they are spatially and temporally more extensive. Moreover, they allow estimations to be made of both pollutant levels and impacts on biological receptors. For this, biomonitoring can directly provide data on the potential effects and integrated toxicities of pollutants to reflect the corresponding degree of deleteriousness in the environment [15].

The release of pollutant HMs into aquatic environments can lead to direct toxic effects on sensitive species through sublethal or lethal impacts. Additionally, certain HMs may transform into persistent, more toxic metal compounds that bioaccumulate in organisms and magnify within the trophic web [15]. When several of them (e.g., Hg, Cr, Cd, Ni, Cu, and Pb) enter water systems, they can prove extremely toxic to aquatic organisms and can cause disruptions at several trophic levels [14]. Among the pollutants, those composed of Cd, Cu, and Cr are not considered the most toxic metals, but rather among the most toxic metals for marine ecosystems. However, it is highlighted that copper in trace amounts is essential to all organisms for a variety of biological processes, but cnidarians like *A. aurita* may be more susceptible to damage by copper than their symbiotic algae [18].

Thus, the main objective of this work is to assess how their presence in a marine environment can affect organisms like cnidarians and their potential preys.

The environmental Cd hazard has been assessed in freshwater as well as in marine and terrestrial ecosystems [19,20]. This HM can be found naturally in water and soil at low concentrations, not only owing to natural processes like volcanic eruptions and crustal erosion, but also to anthropogenic activities, such as mining and smelting, and shifts to aquatic systems via runoff [21]. It subsequently ends up in saline water environments. In industry, Cd is used to manufacture batteries, PVC plastics, and paint pigments. It can also be found in soil given the use of insecticides, fungicides, sludge, and commercial fertilizers by agriculture [14]. This non-essential metal is often toxic even at relatively low concentrations and can cause adverse effects given its high bioaccumulation potential. Cd toxicity for aquatic organisms significantly varies and depends mainly on the concentration of its free ionic form rather than on the concentration of total dissolved Cd [22,23].

Chrome is one of the HMs to which most toxicological importance is attached today. It is present in rocks, plants, soils, animals, fumes, and volcanic gases. Its various effects on organisms’ lives are related to the physico-chemical forms in which it occurs [24].

Its derived compounds are mainly chromates and dichromates, which are used in pigments and dyes, leather tanning, and wood treatment. Industrial effluents containing Cr can eventually reach oceans in different chemical forms influenced by organic matter (OM). If OM is present in large quantities, Cr^6+^ will be reduced to Cr^3+^, which can either be adsorbed in particles or form insoluble complexes [14,25].

The effect of Cu on marine biota largely depends on the amount of free Cu (Cu^2+^) that accumulates in receiving waters. This is determined by the flux of total Cu, its relative solubility, and the concentration of Cu-binding ligands [26]. Of all the natural Cu sources arriving at oceans, wind-blown mineral dust is the largest component to provide seasonal micronutrient pulses to regions that are often limited by such resources [27,28,29]. Emissions resulting from natural fires rank second in non-anthropogenic Cu aerosol production and largely depend on location, with some biomes showing negligible Cu release and others being major regional sources [30].

To assess the toxicity of HMs, common approaches involve utilizing bio-monitoring methods with bio-indicators. These indicators, encompassing species, species groups, or biological communities, offer insights into contamination through real ecosystem observations. Alternatively, laboratory toxicity tests provide an indirect means of inferring environmental quality.

Selecting the right model organism for toxicity tests is critical in applying the bio-indicator model, given that certain organisms are more suitable than others for specific tests, as seen in toxicological impact studies on marine organisms like sea urchins [20,31]. Sea urchin gamete models offer advantages a priori, such as their wide geographical distribution, abundance, and easy collection and maintenance [32]. Sea urchin bio-indicators present challenges, including the requirement of a sufficient number of individuals to ensure both male and female specimens with viable gametes, preventing unintended spawning due to temperature changes during transport, and necessitating a fertilization success rate of at least 90% to proceed with the process [31,32], being impractical for toxicity tests.

To gain a comprehensive understanding of HMs’ toxicity, *Phaeodactylum tricornutum* and the crustacean *Artemia salina* serve as established model organisms, representing primary producers and consumers, respectively. While these models offer valuable insights, there is a lack of secondary consumer models. Addressing this gap, recent research has spotlighted cnidarians as potential indicators of marine environmental conditions owing to their unique sensitivity to stress and swift responsiveness to disturbances [33]. These organisms form part of the gelatinous zooplankton, which includes approximately 2000 widely recognized species [34] as key members of ocean ecosystems. They also play an important role in the organization of marine food webs [35,36,37] as an energy source in both pelagic and deep-sea food webs by supporting C trophic transfer from surface waters to euphotic environments [38,39]. These gelatinous organisms are also active predators that forage on a wide range of prey, from mesozooplankton and ichthyoplankton to microplankton [40,41,42], gelatinous species, and emergent zooplankton [43]. Therefore, jellyfish exert both the top-down and bottom-up control of zooplankton and, indirectly, of phytoplankton communities by cascading effects [44].

Of cnidarians, the jellyfish *Aurelia aurita* is a promising model organism in the ecotoxicology field because it can be used to predict the effects of chemicals and other stressors on the marine environment, such as oil organic chemicals and HMs [45,46,47,48]. It is one of the most abundant and commonest gelatinous zooplankton species in the world. It is an epipelagic scyphozoan with a cosmopolitan distribution that is located in the waters along the neritic zone [47]. Its biological cycle is complex because it combines a sessile asexual polyp phase and a free-living sexual medusa phase [49]. Worldwide *A. aurita* populations are defined by their high diversity, characteristic life cycle, abundance, growth, strobilation timing and periodicity, time and size upon sexual maturation, and jellyfish longevity [50]. In nature, strobilation, the process by which the free-living phase is generated, is a seasonal process that starts in winter or early in spring [50,51]. In winter, water temperature lowers and acts as an environmental signal, which is perceived by polyps. A single colony of polyps can asexually produce genetically identical male or female jellyfish [52,53], which is a huge advantage when conducting studies because it avoids intra- and interpopulation variation problems [54].

Of all mentioned above, *A. aurita* stands out as a valuable model organism thanks to its ease of laboratory handling, with the ability to readily produce ephyrae from polyps through a simple heat shock method involving controlled temperature changes over a brief period, alongside uncomplicated maintenance requirements.

Therefore, to broaden our knowledge about the effects of Cd, Cu, and Cr pollution at different marine trophic levels and specifically in organisms for which their effects have not yet been tested, such as *A. aurita*, the present study poses the following hypotheses: (a) the toxicity of elements is different at the trophic levels they affect; (b) toxicants cause damage during acute exposure.

This work also attempts to support the ephyra of the scyphomedusa *A. aurita* as a model for marine ecotoxicological assays to make progress in the study of substances introduced into the natural environment and to understand the consequences of this.

## 2. Materials and Methods

In order to study organisms’ resilience to the introduction of pollutants, three bioassays were performed based on aquatic organisms’ reaction to a wide range of pollutants [55].

The toxic reference salts selected to study the effects of Cd, Cu, and Cr were cadmium nitrate Cd(NO_3_)_2_, copper nitrate Cu(NO_3_)_2_, and potassium dichromate K_2_Cr_2_O_7_, respectively. All salts (analytical grade) were supplied by Sigma-Aldrich™ (Darmstadt, Germany). This study was carried out with three marine species used as bioindicators of marine toxicity of HMs. The test protocol carried out on each is provided below.

### 2.1. Toxicity Test with Phaeodactylum tricornutum

The microalgae test was carried out according to standardized protocol Algaltoxkit M™ (Marine Toxicity Test with Microalgae) developed by Microbiotests Inc. (Gent, Belgium). The kit follows Standard ISO 10253:2017. This is an algal growth inhibition test performed in vials with the marine diatom *Phaeodactylum tricornutum*. The concentrations were 0.01, 0.03, 0.06, 0.12, and 0.25 mg/L and they were incubated for 3 days at 20 °C (±2 °C) with constant and uniform lighting provided by cool white fluorescent lamps. Lighting was 10,000 lux for the side of the long cell or 3000–4000 lux for the bottom lighting. Growth was monitored by optical density (OD) measurements in a spectrophotometer equipped with a 670 nm filter and a 10 cm cell holder. ODs were converted into algal numbers with the help of the “Optical Density/Number of Algae” (OD/N) sheet included in each Algaltoxkit M™. After culturing, microalgae growth measurements were monitored for 24 h, 48 h, and 72 h. For each treated concentration and all of the used toxicants (cadmium nitrate, copper nitrate, and potassium dichromate), tests were independently conducted in triplicate.

### 2.2. Toxicity Test with Artemia salina

The toxicity test was carried out according to standardized protocol Artoxkit M™ (Artemia Toxicity Screening Test for Estuarine and Marine Waters) developed by Microbiotests Inc. (Gent, Belgium). Cysts were allowed to hatch during incubation under aerated conditions with constant light (light source of 3000–4000 lux) at 25 °C in standard seawater. Hatching started after about 18–20 h. After 30 h, most larvae had moulted in the instar II–III stage. Briefly, 2 mL of filtered seawater and 10 Artemia larvae were added to each well. The mixture concentrations were 6.25, 12.5, 25, 50, and 100 mg/L for each employed toxicant. After the 24 h and 48 h treatments at each metal concentration, the numbers of living and dead Artemia nauplii were counted. Dead individuals were determined if no movement of appendages was observed within 10 s. For every treated concentration, and for all of the employed toxicants (cadmium nitrate, copper nitrate, and potassium dichromate), tests were conducted in four replicates.

### 2.3. Toxicity Test with Aurelia aurita

This assay followed the guidelines of the test previously carried out by Faimali et al. [48]. Toxicity tests were prepared using the *A. aurita* ephyra collected immediately after strobilation (0 days of age). The ephyra used in these experiments were obtained directly from the polyps reared in the Oceanogràfic de València laboratories (València, Spain).

Two end-points were evaluated: one was frequency of pulsation, defined as the number of pulsations performed by ephyra within a defined time unit (30 s), measured as the % alteration of pulsations (compared with the control). The second end-point was to measure the % mortality of ephyra for each concentration (compared with the control).

Ephyra were placed three by three in a multiwell plate containing 10 mL of solution. The applied concentrations were 1.9, 3.75, 7.5, 15, and 30 mg/L for each utilized salt (the corresponding Cd, Cu, and Cr concentrations found in each salt appear in Table 1). Plates were sealed and left in the thermostatic room at 20 °C in the dark. After 24 h and 48 h, the beats per minute of all of the ephyra and % mortality were evaluated. For each treated concentration, and for all the used toxicants (cadmium nitrate, copper nitrate, and potassium dichromate), tests were conducted in five replicates.

### 2.4. Statistical Analysis

For the three organisms used in the tests, EC_50_ (half maximal effective concentration) was obtained using a Probit analysis using the SPSS™ S statistical package (v20, IBM). EC_50_ was calculated after 24 h and 48 h for *A. salina* and *A. aurita* and after 72 h for *P. tricornutum*. For the three tested organisms, the statistical significance of the differences between means and groups (*p* < 0.05) was estimated based on a one-way ANOVA and a Student’s *t*-test using SPSS™ (v20, IBM, Armonk, NY, USA) and MS Excel™ (v17.0. Microsoft Inc., Redmond, WA, USA).

## 3. Results

### 3.1. Phaeodactylum tricornutum (Primary Producer Model)

**Figure 1:**
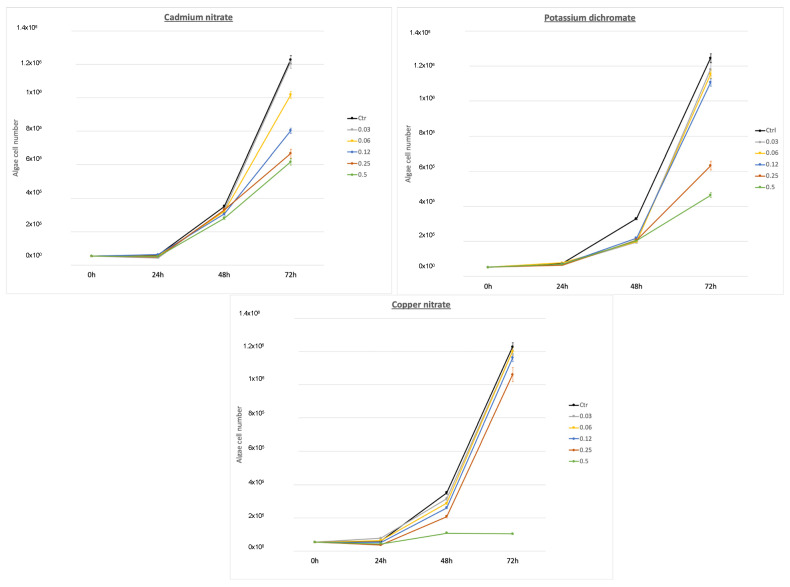
Effect of Cu(NO_3_)_2_, K_2_Cr_2_O_7_, and Cd(NO_3_)_2_ on *Phaeodactylum tricornutum* growth at several concentrations measured at 72 h (*t*-test, *p* < 0.05). Data are for *P. tricornutum* algae (means ± standard error, n = 3).

In order to observe the effect of Cu, Cr, and Cd on *P. tricornutum* growth, algae were exposed to several concentrations of salts and their growth rate was assessed (Figure 1). The *P. tricornutum* samples received 0.03, 0.06, 0.12, 0.25, and 0.5 mg/L of each salt (Cd(NO_3_)_2_, Cu(NO_3_)_2_, and K_2_Cr_2_O_7_). Growth was determined after 72 h (the corresponding Cd, Cu, and Cr concentrations on each salt are shown in Table 1). The results of the study revealed significant inhibitory effects of different concentrations of cadmium nitrate, potassium dichromate, and copper nitrate on the growth of the algae. When compared with the control group, a concentration of 0.03 of cadmium nitrate exhibited a growth inhibition of 1.22%, while the concentration of 0.03 of potassium dichromate showed an inhibition of 5.3%. Notably, the inhibition increased with higher concentrations, with the concentration of 0.06 of cadmium nitrate resulting in a growth inhibition of 17% and the concentration of 0.06 of potassium dichromate showing an inhibition of 7.54%. Moreover, the concentration of 0.25 of cadmium nitrate exhibited a substantial inhibition of 34.5%, whereas the concentration of 0.25 of potassium dichromate displayed a remarkable inhibition of 50.6%. The highest inhibitory effects were observed at the concentration of 0.5, with cadmium nitrate inhibiting growth by 49.7% and potassium dichromate showing an inhibition of 64.6%. Comparatively, copper nitrate exhibited relatively lower inhibitory effects, with the concentration of 0.03 resulting in a growth inhibition of 2.05% and the concentration of 0.06 showing an inhibition of 2.18%. However, as the concentration increased, so did the inhibitory effects, with the concentration of 0.12 resulting in a growth inhibition of 5.39%, the concentration of 0.25 exhibiting an inhibition of 13.59%, and the concentration of 0.5 demonstrating the highest inhibition of 91.62%. These findings indicate that, among the three substances tested, cadmium nitrate and potassium dichromate exerted stronger inhibitory effects on growth compared with copper nitrate Therefore, among the three HMs tested, the most toxic HMs for *P. tricornutum* were Cr and Cd followed by Cu at 72 h and the highest concentration.

The results of the study revealed the EC_50_ values for the algae *P. tricornutum*, indicating the concentration at which 50% inhibition of growth occurred. EC_50_ for potassium dichromate was determined to be 15.3 mg/L. In contrast, cadmium nitrate exhibited a lower EC_50_ value of 2.49 mg/L, suggesting a higher toxicity to the algae. Similarly, copper nitrate demonstrated a relatively lower EC_50_ value of 1.205 mg/L, indicating a strong inhibitory effect on the growth of *P. tricornutum*. These findings highlight the varying toxicities of the tested compounds, with cadmium nitrate and copper nitrate showing lower toxicity compared with potassium dichromate towards *P. tricornutum* (Table 2).

### 3.2. Artemia salina (Primary Consumer Model)

**Figure 2:**
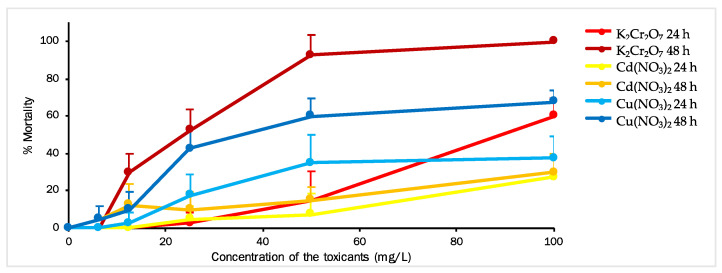
The effect of Cu(NO_3_)_2_, K_2_Cr_2_O_7_, and Cd(NO_3_)_2_ on the mortality of *Artemia salina* at various concentrations measured at 24 and 48 h (*t*-test, *p* < 0.05). Data are for *Artemia* larvae (means ± standard error, n = 4).

To investigate the impact of Cu, Cr, and Cd on the mortality of *A. salina*, the crustaceans were exposed to different concentrations of these HM salts, and their mortality rates were assessed (Figure 2). The concentrations of each salt used for the larvae ranged from 6.25 to 100 mg/L. Mortality was evaluated after 24 h and 48 h of exposure.

Based on the results obtained after 24 h, Artemia larvae showed low sensitivity to Cr and Cd concentrations up to 25 mg/L, as well as to Cu concentrations up to 12.5 mg/L. The highest mortality rate during de first 24 h was 60% for K_2_Cr_2_O_7_, followed by 37.5% for Cu(NO_3_)_2_ and 27.5% for Cd(NO_3_)_2_ (*t*-test, *p* < 0.05). The calculated EC_50_ values for Cu(NO_3_)_2_, K_2_Cr_2_O_7_, and Cd(NO_3_)_2_ after 24 h were 95.773, 91.359, and 150.167 mg/L, respectively (Table 2).

After 48 h of exposure, significant increases in *Artemia* mortality were observed at concentrations as low as 6.25 mg/L for Cr and 12.5 mg/L for the other two toxicants. The mortality rate for Cd(NO_3_)_2_ remained consistent at both exposure times. The highest mortality rate (100%) was recorded for *A. salina*, indicating that Cr was the most toxic metal, followed by 67.5% for Cu and 30% for Cd (*t*-test, *p* < 0.05). The calculated EC_50_ values for Cu(NO_3_)_2_, K_2_Cr_2_O_7_, and Cd(NO_3_)_2_ after 48 h were 37.201, 23.554, and 153.840 mg/L, respectively (Table 2).

### 3.3. Aurelia aurita (Secondary Consumer Model)

**Figure 3:**
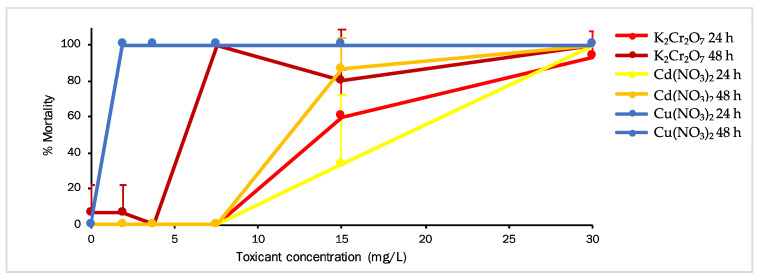
The effect of Cu(NO_3_)_2_, K_2_Cr_2_O_7_, and Cd(NO_3_)_2_ on *Aurelia aurita* mortality at several concentrations measured at 24 h and 48 h (*t*-test, *p* < 0.05). Data are for *A. aurita* ephyra (means ± standard error, n = 5).

In order to note the effect of Cu, Cr, and Cd on *A. aurita* mortality, ephyras were treated with five concentrations of the studied toxicants, and their mortality rate was assessed (Figure 3). For the *A. aurita* ephyra, the ranges of concentrations of each toxicant were 1.9, 3.75, 7.5, 15, and 30 mg/L. After that, the results at 24 h revealed that the ephyras were not affected by K_2_Cr_2_O_7_ and Cd(NO_3_)_2_ at concentrations up to 7.5 mg/L, but mortality was 100% at the lowest concentration (1.9 mg/L) with Cu(NO_3_)_2_. At the highest exposure concentration, 100% mortality was caused by Cu and Cd, followed by 93.33% mortality by Cr (*t*-test, *p* < 0.05). The calculated EC_50_ values of Cu(NO_3_)_2_, K_2_Cr_2_O_7_, and Cd(NO_3_)_2_ at 24 h were 0.283, 16.571, and 19.880 mg/L, respectively (Table 2). The results at 48 h of exposure showed that the *A. aurita* ephyras exposed to Cu(NO_3_)_2_ followed the same pattern as the measurements taken at 24 h with 100% mortality at all concentrations. With K_2_Cr_2_O_7_, mortality started to significantly increase from 3.75 mg/L, and from 7.5 mg/L for Cd(NO_3_)_2_. Mortality at 48 h for the highest concentration was 100% for all three studied toxicants (*t*-test, *p* < 0.05). The EC_50_ values calculated for Cu(NO_3_)_2_, K_2_Cr_2_O_7_, and Cd(NO_3_)_2_ at 48 h were 0.283, 6.726, and 12.343 mg/L, respectively (Table 2).

**Figure 4:**
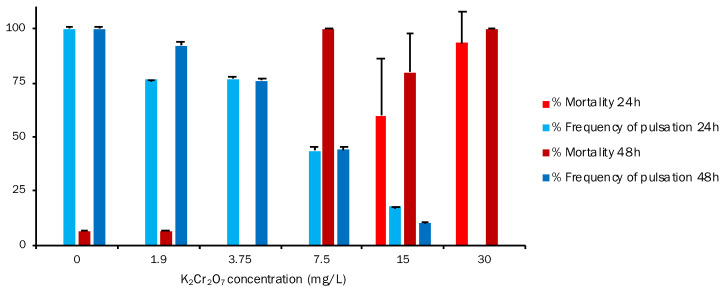
Alteration of frequency of pulsation (%Fp) and mortality (% M) of *A. aurita* ephyra after the 24 h and 48 h exposures to increasing potassium dichromate concentrations (one-way ANOVA, *p* < 0.05). Data are for *A. aurita* ephyra (means ± standard error, n = 5).

This study also involved toxicity screening with the three different reference toxic substances. Two end-points were considered: frequency of pulsation (Fp) and mortality (M); the percentage of alteration to Fp (%Fp) and the percentage of mortality (%M) were calculated at the 24 h and 48 h exposure times, and were compared to the untreated control. The results obtained for exposing the 0-day-old ephyra to different concentrations of potassium dichromate are reported in Figure 4. This compound had a significant effect on both end-points. %Fp for both 24 h and 48 h showed an inverse correlation with a rising toxicant concentration, and was 0% at the highest concentration (30 mg/L). Mortality at 24 h was 93.33%, with 100%M at 48 h for the high concentration. Regarding the values obtained for %M at 24 h, the LOEC (lowest observed effect concentration) was 15 mg/L. For %M at 48 h, an incongruent value was observed because the LOEC was 0 mg/L in the untreated control. The same percentage of mortality as the control was obtained at the lowest K_2_Cr_2_O_7_ concentration, and only 1 ephyra of the 15 exposed ones was death. No mortality took place at a concentration of 3.75 mg/L, but at the next concentration (7.5 mg/L), mortality was 100% compared with 0%M at the same concentration after 24 h. M% at 48 h at the remaining concentrations (15 mg/L and 30 mg/L) was 80% and 100%, respectively. In this assay, Fp was the most sensitive end-point (in magnitude of response and data reliability terms) compared with mortality.

**Figure 5:**
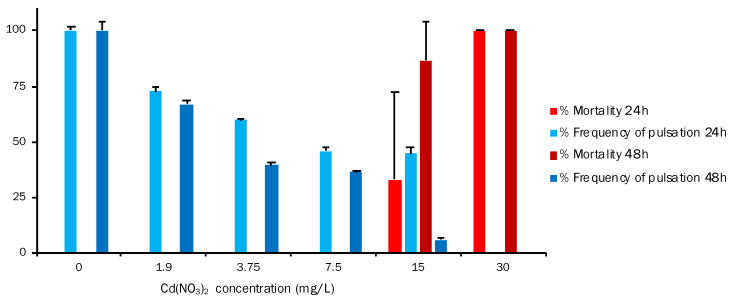
Alteration of frequency of pulsation (%Fp) and mortality (% M) of *A. aurita* ephyra after the 24 h and 48 h exposures to increasing cadmium nitrate concentrations (one-way ANOVA, *p* < 0.05). Data are for *A. aurita* ephyra (means ± standard error, n = 5).

**Figure 6:**
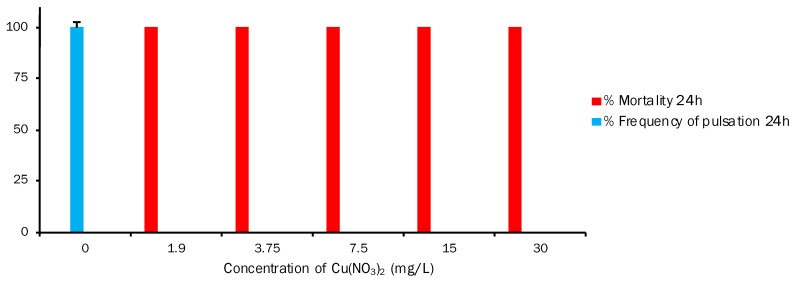
Alteration of frequency of pulsation (%Fp) and mortality (% M) of *A. aurita* ephyra after the 24 h and 48 h exposures to increasing copper nitrate concentrations (one-way ANOVA, *p* < 0.05). Data are for *A. aurita* ephyra (means ± standard error, n = 5).

The results of exposing ephyra to different cadmium nitrate concentrations are shown in Figure 5. Like Cr, this compound also had a significant effect on both end-points. %Fp at both 24 h and 48 h lowered when the toxicant concentration rose, and was 100% at 0 mg/L and 0% at 30 mg/L. This also implies, as we mentioned above, an inverse correlation between %Fp and %M, which was observed at 15 mg/L and at both 24 h and 48 h. Mortality increased and, therefore, %Fp decreased (24 h: 33.33%M and 44.79%Fp; 48 h: 86.66%M and 6.17%Fp). Regarding the LOEC value for %M, it is 15 mg/L at both exposure times, with 33.33%M and 86.66%M at 24 h and 48 h, respectively. At 30 mg/L, mortality was 100% at both exposure times. The results obtained for exposing ephyra to different copper nitrate concentrations are shown in Figure 6. This graph depicts a different situation to those previously described. For Fp, the values were 100% at 24 h in the untreated control and 0% at the five different reference toxic concentrations chosen for the test. In mortality terms, the mortality of the exposed *A. aurita* ephyras was 0% in the control, but 100% at the different toxic concentrations. The obtained results showed that Cu significantly affected ephyras from the lowest tested Cu(NO_3_)_2_ concentration (1.9 mg/L) to the highest one (30 mg/L).

## 4. Discussion

**Figure 7:**
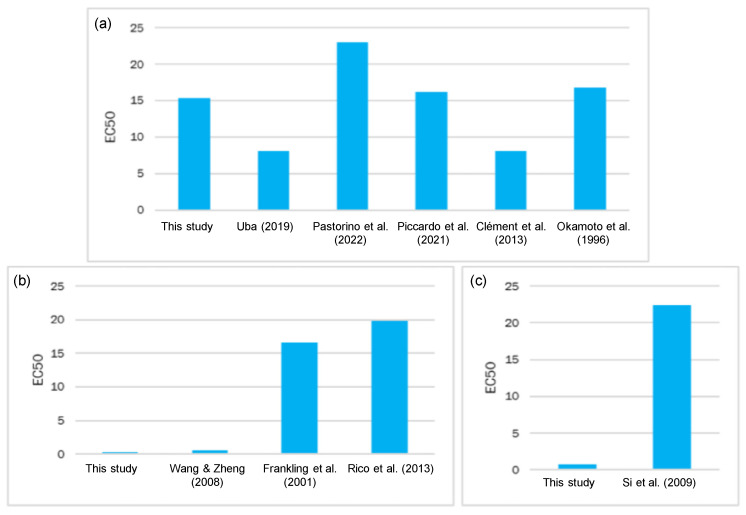
Comparison of the EC_50_ values (mg/L) of *Phaeodactylum tricornutum* from other studies to the present study: (**a**) the EC_50_ values of potassium dichromate at 72 h; (**b**) the Cu values at 72 h; (**c**) the Cd values at 72 h [56,57,58,59,60,61,62,63,64].

Regarding Cr toxicity for the marine algae, our results (Table 2) showed an EC_50_ ± SD of 15.378 ± 7.081 mg/L for potassium dichromate at 72 h. However, other authors report different values (see Figure 7). In the study of Uba [56], K_2_Cr_2_O_7_ obtained an EC_50_ value of 8.07 ± 0.03 mg/L, while the R^2^ value was 0.99. Other authors have studied how species’ sensitiveness to the same chemicals can vastly vary [57]. They characterized the non-standardized diatom *Chaetoceros tenuissimus* by growth inhibition, biochemical, and infrared-spectroscopy (FT-IR) tests to compare the results to the standardized diatom *Phaeodactylum tricornutum*. The two species were exposed for 72 h to four chemicals: nanoparticles (n-TiO_2_, n-ZnO), potassium dichromate, and surfactant (polyethylene glycol, PEG). The obtained EC_50_ ± SD (mg/L) for *P. tricornutum* and *C. tenuissimus* were 22.97 ± 1.34 and 19.84 ± 1.45, respectively [57]. Other authors have evaluated the impacts of 16 different leachates of plastic-made packaging on marine species from different trophic levels (bacteria, algae, and echinoderms) [58]. The results obtained in that study evidenced that the tested doses were unable to significantly affect bacteria (*Vibrio fischeri*) and algae (*P. tricornutum*). Algae responses were measured by K_2_Cr_2_O_7_ (EC_50_ = 16.21 ± 1.72 mg/L). Another ecotoxicity test [59] has studied the toxicity of metal aqueous suspensions to microcrustaceans *Daphnia magna* (72 h exposure), algae *P. tricornutum* (72 h growth inhibition), and rotifer *Brachionus plicatilis* (48 h exposure). EC_50_ calculated for algae at 72 h was 8.11 mg/L. Pastorino et al. [57] has demonstrated that EC_50_ for *P. tricornutum* after 72 h of exposure to K_2_Cr_2_O_7_ fell within the 16.76–20.84 mg/L interval. According to our results, EC_50_ obtained for K_2_Cr_2_O_7_ came quite close to the reference values of other papers, which indicates that this alga resisted Cr more than other HMs.

On Cd toxicity for the marine algae, our results for cadmium nitrate indicated an EC_50_ ± SD (mg/L) of 2.494 ± 2.494 at 72 h (Table 2). Some authors reported that, at 72 h, the Cd EC_50_ value for *P. tricornutum* was as high as 22.39 mg/L, which reveals its excellent tolerance to Cd. Compared with other microalgae species, they reported lower EC_50_ values: 1.87 µg/L of Cd for *Scenedesmus quadricauda*, 2.13 µg/L for *Aulacoseira granulate*, and 1.8 mg/L for *Teraselmis gracilis* [60,61]. According to our results, EC_50_ for Cd(NO_3_)_2_ was far-removed from the reference values of the aforementioned papers. Hence, further research into exposing *P. tricornutum* to Cd must be conducted.

On Cu toxicity for the marine algae *P. tricornutum*, we obtained an EC_50_ ± SD (mg/L) of 1.205 ± 0.322 at 72 h for Cu(NO_3_)_2_ (Table 2). Population growth started to be affected at a concentration of 0.06 mg/L (Figure 1). Wang and Zheng [62] observed that, at the Cu^2+^ concentration of 0.32 μg/mL, *P. tricornutum* cell density was significantly lower than that in the control (*t*-test, *p* > 0.05). EC_50_ for Cu^2+^ at 72 h for this alga was calculated using a regression analysis and was 0.565 μg/mL. In that study, Cu at lower concentrations (<0.2 μg/mL) did not have any obvious adverse effect on *P. tricornutum* population reproduction, but Cu significantly inhibited this diatom’s reproduction at >0.32 μg/mL. This finding indicates that >0.32 μg/mL Cu^2+^ concentrations exceed the safety concentration level for this alga. In previous studies, Jung et al. [63] exposed marine algae *Nitzschia pungens* to several antifouling biocides, including copper pyrithione. EC_50_ (μg/L) of this compound recorded at 96 h was 0.319 ± 0.016. Franklin et al. [64] studied Cu toxicity in *P. tricornutum*. The EC_50_ values at 48 h and 72 h were 142 ± 47 and 158 ± 63 nmol/L, respectively, and complete growth inhibition occurred at 11.8 μmol/L. The cell light scatter properties of *P. tricornutum* depended on cell size and intracellular granularity. Franklin et al. [64] noted how Cu brought about an increase in cell size after 24 h of exposure, with 50% of cells being larger than the controls at 3.15 μmol/L, and similar increases in cell size observed after 48 h and 72 h at 157.5 nmol/L and an EC_50_ value of 126 ± 47 nmol /L. Similar changes in cell size and granularity were also reflected in side-angle light scatter changes, with an EC_50_ value of 189 nmol/L after 48 h and 72 h of Cu exposure [65]. In line with our EC_50_ results, these values differ from other studies (Figure 7). Hence, more research is required to collect more reliable data. Therefore, according to our EC_50_ results for each salt (Table 2) and the corresponding concentration of each metal on its respective salt (Table 1), we conclude that the relative toxicity for *P. tricornutum* is Cu > Cd > Cr.

The brine shrimp *A. salina* is a suggested organism for bioassays because it functions like other zooplankton crustaceans, which accumulate trace elements and then transfer them to a higher trophic level [66]. *Artemia* has been used to study metal toxicity in other studies [67], some of which have demonstrated that brine shrimp is moderately sensitive to a wide range of metals [31,68,69,70].

On Cr toxicity for the crustacean *A. salina*, our results for K_2_Cr_2_O_7_ showed an EC_50_ ± SD (mg/L) of 91.359 ± 6.746 at 24 h and a value of 23.554 ± 2.383 at 48 h (Table 2). Kalčíková et al. [71] tested *A. nauplii* immediately after hatching, called the first instar, and the mean 24 h EC_50_ of K_2_Cr_2_O_7_ was 39.7 mg/L (n = 8) with SD = 10.2 mg/L. For the second and third instars, which were tested 24 h after hatching, the mean 24 h EC_50_ of K_2_Cr_2_O_7_ was lower (27.9 mg/L (n = 8)) and SD was 5.1 mg/L, which revealed the test’s lower variability.

The test using the second and third *Artemia* instars was assessed because, the greater the sensitivity, the lower the variability. It was, therefore, used for other toxicity testing. In another study carried out in 2012 by Umarani et al. [72], acute Cr toxicity at 96 h to adult and subadult Artemia exposed to different salinity conditions was tested: at 40, 60, and 80 ppt salinity, the EC_50_ values for subadult Artemia were 0.519, 0.784, and 1.192 mg/L, respectively, while the EC_50_ values for adult Artemia were 1.031, 0.413, and 0.887 mg/L, respectively. In another study, Eduardo et al. [73] determined that EC_50_ was variable in different development stages. In the first 24 h after hatching, EC_50_ was one of the highest found in that study (21 μg/mL); it then decreased to 15 μg/mL in the 2nd stage and remained unchanged until the 5th stage. There were no significant differences. In the 6th and 7th stages, EC_50_ increased to 21 μg/mL, with a further rise to 25 μg/mL in the 8th stage, which represented the peak EC_50_ value in that study. A lowering EC_50_ trend occurred in the 10th and 11th stages, with values of 21 μg/mL and 16 μg/mL, respectively. From the 12th to the 15th stage, the EC_50_ values did not significantly differ from one another. In another paper, acute K_2_Cr_2_O_7_ toxicity in *A. salina* larvae was studied as an alternative method to be applied to ecotoxicology. In this versatile method, 24 h nauplii were exposed to different concentrations of the compound, and EC_50_ was 12.5 mg/L [26]. According to the published papers on Artemia toxicity to K_2_Cr_2_O_7_, the EC_50_ values moderately differed from those obtained herein (Figure 7). However, they all showed the toxicity of this compound for *Artemia* larvae.

**Figure 8:**
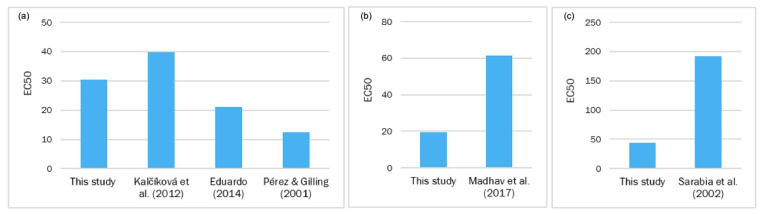
Comparison of the EC_50_ values (mg/L) of *Artemia salina* from other studies to those in the present study: (**a**) the EC_50_ values of Cr at 24 h; (**b**) the Cu values at 24 h; (**c**) the Cd values at 24 h [25,70,71,73,75].

On Cd toxicity for the crustacean, our results for Cd(NO_3_)_2_ gave an EC_50_ ± SD (mg/L) of 150.167 ± 27.496 at 24 h and a value of 153.840 ± 65.674 at 48 h (Table 2). Previous studies showed that *Artemia* was among those crustaceans that were most tolerant to Cd toxicity, as shown by the 24 h EC_50_ values corresponding to the different studied species and populations. They ranged from 98 mg/L to 286 mg/L compared with the 48 h EC_50_ (0.5–17 mg/L) reported for other crustaceans [70,74]. This tolerance can be partly explained by the marked effectiveness of Cd for metallothionein induction in *Artemia* [70]. Hadjispyrou et al. [24] reported that the EC_50_ value for cadmium chloride to cause 50%M in *Artemia* was 155.5 mg/L at 24 h, with a 95% confidence interval (95% CI) of 148.8–162.5 mg/L. According to the bibliography and the obtained EC_50_ values (Figure 8), we determined that *A. salina* was well tolerant to cadmium nitrate because the EC_50_ value was over 98 mg/L in all cases.

On Cu toxicity for *A. salina*, our Cu(NO_3_)_2_ results gave an EC_50_ ± SD (mg/L) of 95.773 ± 28.284 at 24 h and a value of 37.201 ± 1.872 at 48 h (Table 2). Madhav et al. [75] carried out a study in 2017 and chose a range of concentrations from 25 to 800 mg/L to run toxicity experiments with adult *Artemia*. The EC_50_ value was 61.4 mg/L (95% CI of 47.4–83.4 mg/L) and 35.75 mg/L (95% CI of 30–42 mg/L) at 24 h and 48 h, respectively. Our results showed that *Artemia* quite well tolerated copper nitrate up to concentrations of around 60 mg/L (Figure 8). Therefore, according to our EC_50_ results for each salt (Table 2) and the corresponding concentration of each metal on its respective salt (Table 1), we conclude that the relative toxicity for *A. salina* is Cr > Cu > Cd.

**Figure 9:**
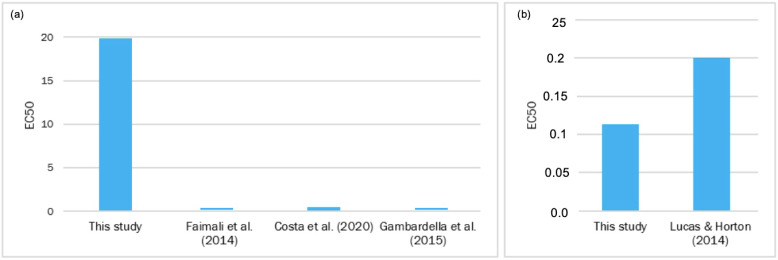
Comparison of the EC_50_ values (mg/L) of *Artemia salina* from other studies to those of the present study: (**a**) the EC_50_ values of Cr at 24 h; (**b**) the Cu values at 24 h. [32,45,48,76].

Regarding Cd toxicity for the *A. aurita* ephyra, our results for Cd(NO_3_)_2_ gave an EC_50_ ± SD (mg/L) of 19.880 ± 5.519 at 24 h and a value of 12.343 ± 2.588 at 48 h (Table 2). According to the Fp results (Figure 5), at both 24 h and 48 h, when mortality increased, %Fp dropped (24 h: 33.33%M and 44.79%Fp; 48 h: 86.66%M and 6.17% Fp). The LOEC value for %M is 15 mg/L at both exposure times. In the study conducted by Faimali et al. [48], after 24 h of exposure, cadmium nitrate had an effect on both end-points (acute and sublethal), as evidenced by the 0.5 mg/L concentration for immobilization and that of 0.1 mg/L for Fp. After 48 h, the same effects were caused by the lower concentration of 0.05 mg/L for both end-points. It should be highlighted that both end-points showed 100% response at 1 mg/L after 24 h of exposure. From the obtained data (EC_50_) on the effect of cadmium nitrate on *A. aurita*, we obtained 0.07 mg/L at 24 h and 0.13 mg/L at 48 h. The comparison of EC_50_ with *A. aurita* showed that the new biological model appeared to be the most sensitive of the considered model organisms [48]. Costa et al. [45] exposed Aurelia sp. ephyras to different 1–4 μm microplastics (MPs). A relatively and slightly significant difference in effect terms (immobility, Fp) in treatments was observed. A difference in sensitivity in the end-points for LOEC, Fp, and EC_50_ after 24 h was noted. These results showed that the behavioral end-point (Fp) was more sensitive than the acute one (immobility) (LOEC Fpn = 0.01 mg/L versus LOEC immobility = 0.1 mg/L) for all of the exposure conditions. Conversely, after 48 h, MPs had significantly affected (*p* < 0.05) both end-points at the lowest tested concentration. A toxic effect was also observed, but only for immobility in EC_50_ terms at both exposure times and independently of the exposure conditions. The EC_50_ values at 24 h for immobility and Fp were 0.40 and 0.13 mg/L, respectively. Overall, the behavioral end-point (Fp) was very sensitive because a significant effect was noted at the lowest tested concentration (0.01 mg/L) [44]. In another study, Gambardella et al. [32] investigated the potential toxicity of Ag-NPs (silver nanoparticles) for the marine ecosystem by analyzing effects on several organisms belonging to different trophic levels. Algae (*Dunaliella tertiolecta* and *Skeletonema costatum*), cnidaria (*A. aurita* jellyfish), crustaceans (*Amphibalanus amphitrite* and *Artemia salina*), and echinoderms (*Paracentrotus lividus*) were exposed to Ag-NPs and different end-points were evaluated. The results showed that all of the end-points were able to underline a dose-dependent effect. Jellyfish were the most sensitive species, followed by barnacles, sea urchins, green algae, diatoms, and brine shrimps. The comparison of EC_50_ to the selected species highlighted that jellyfish appeared to be the most sensitive model organisms of all of those investigated: EC_50_ was 0.09 with 0.15 mg/L of cadmium nitrate at 24 h and 48 h, respectively. When considering previous ephyra studies [48], we find that the EC_50_ and LOEC values for the mortality and Fp of *A. aurita* are lower than those obtained herein (Figure 9). However, a correlation appeared between the mortality and Fp end-points, and ephyra responded efficiently to the selected range of toxicant concentrations.

On Cu toxicity for the *A. aurita* ephyra, our results for Cu(NO_3_)_2_ gave an EC_50_ ± SD (mg/L) of 0.283 ± 0 t 24 h and a value of 0.283 ± 0 at 48 h (Table 2). In mortality terms, the exposed *A. aurita* ephyras presented 0%M in the control, but 100%M at the different toxic concentrations (Figure 6). Lucas and Horton [76] studied the short-term effects of HMs (including Cu) on the polyps of the common jellyfish *A. aurita*. They examined the independent effects of Cu on polyp condition aspects, including budding, strobilation, deformities, and mortality. The results showed that 200 μg Cu/L exceeded polyps’ tolerance to this metal and rapidly led to mortality. For all of the treatments with a high Cu concentration (200 μg/L), polyp mortality was 89.5 ± 8.3% by day 4, reaching 100%M by day 17. In another study, Karntanut and Pascoe [77] examined the comparative sensitivity of three Hydra species to three important metal pollutants: Cu, Cd, and Zn. The selected species were *Hydra vulgaris*, *Hydra viridissima*, and *Hydra oligactis*. The acute toxicity data indicated a similar response of all of the species to each metal, with Cu being the most toxic and Zn being the least toxic. The range of 96 h EC_50_ for Cu, Cd, and Zn for all of the Hydra species was 0.025–0.084 mg/L, 0.16–0.52 mg/L, and 11–14 mg/L, respectively. According to our data and the data observed in the above-cited studies (Figure 9), the conclusion is that Cu, and thus Cu(NO_3_)_2_, is highly toxic for cnidarians at very low concentrations.

As for Cr toxicity for the *A. aurita* ephyra, our results for K_2_Cr_2_O_7_ showed an EC_50_ ± SD (mg/L) of 16.571 ± 4.246 at 24 h and a value of 6.726 ± 2.004 at 48 h (Table 2). %Fp at both 24 h and 48 h showed an inverse correlation with an increasing toxicant concentration, with 0% at the highest concentration (30 mg/L), 93.33%M at 24 h, and 100%M at 48 h. According to the values obtained for %M at 24 h, the LOEC was 15 mg/L. For %M at 48 h, an incongruent value was observed because the LOEC was 0 mg/L in the untreated control. Unfortunately, we did not find any paper in which cnidarians were exposed to Cr to compare them. However, we noted that %M and %Fp followed a similar pattern to that of Cd(NO_3_)_2_ (Figure 8), which demonstrates that *A. aurita* is very sensitive to these metals. Therefore, according to our EC_50_ results for each salt (Table 2) and the corresponding concentration of every metal on its respective salt (Table 2), it can be concluded that the relative toxicity for *A. aurita* is Cu > Cr > Cd.

## 5. Conclusions

The global increase in anthropogenic activities in relation to industrial development implies that ever-growing quantities of HMs enter the marine environment through effluents. This calls for continuous monitoring to control or limit their emission levels. For this purpose, the toxicity of and impact on different marine trophic levels need to be considered and deduced. The present study is a good start in the risk assessment and marine environmental conservation quest. This experimental work also allowed us to support other studies that have proposed *A. aurita* as a model for ecotoxicity tests, because our experiments enabled us to identify two end-points (sublethal and acute) with different sensitivity levels. The comparison of the EC_50_ values obtained herein for the three reference toxicants indicates that jellyfish are a very promising model organism for ecotoxicological research purposes. Given the hypotheses posed in this study, and having performed the ecotoxicological test for three organisms, it can be assumed that the toxicity of the three elements is different at the trophic levels they affect and all of the toxicants cause damage during acute exposure. However, algae together with *A. aurita* ephyra have a lower EC_50_. Thus, they could support the jellyfish as a proper model for marine ecotoxicological assays in order to better evaluate the toxicity in higher trophic levels.

## Figures and Tables

**Table 1:** Equivalent concentration values of Cu, Cr, and Cd, all values are given in mg/L.

Organism	Salt Concentration ^1^	Cu	Cr	Cd
*P. tricornutum*	0.03	0.062	0.109	0.001
	0.06	0.068	0.119	0.009
	0.12	0.08	0.139	0.027
	0.25	0.107	0.182	0.065
*A. salina*	6.25	1.322	2.174	1.81
	12.5	2.587	4.249	3.627
	25	5.119	8.399	7.262
	50	10.181	16.699	14.532
	100	20.306	33.299	29.072
*A. aurita*	1.9	0.441	0.73	0.545
	3.75	0.813	1.344	1.083
	7.5	1.575	2.589	2.173
	2.173	3.094	5.079	4.354
	30	6.131	10.059	8.716

^1^ corresponding to all of their salt concentration ranges of (Cu(NO_3_)_2_, K_2_Cr_2_O_7_, and Cd(NO_3_)_2_, respectively).

**Table 2:** The EC_50_ values of *P. tricornutum* at 72 h and the EC_50_ values of *Artemia* larvae and *A. aurita* ephyra at 24 h and 48 h for each toxicant.

Organism	Toxicant	Exposure Time (h)	EC_50_ (mg/L) (Mean ± SD)
*Phaeodactylum tricornutum*	K_2_Cr_2_O_7_	72	15.378 ± 7.081
	Cd(NO_3_)_2_	72	2.494 ± 2.494
	Cu(NO_3_)_2_	72	1.205 ± 0.322
*Artemia salina*	K_2_Cr_2_O_7_	24	91.359 ± 6.746
		48	23.554 ± 2.383
	Cd(NO_3_)_2_	24	150.167 ± 27.496
		48	153.840 ± 65.674
	Cu(NO_3_)_2_	24	95.773 ± 28.284
		48	37.201 ± 1.872
*Aurelia aurita*	K_2_Cr_2_O_7_	24	16.571 ± 4.246
		48	6.726 ± 2.004
	Cd(NO_3_)_2_	24	19.880 ± 5.519
		48	12.343 ± 2.588
	Cu(NO_3_)_2_	24	0.283 ± 0
		48	0.283 ± 0

## Data Availability

Oscar Andreu-Sánchez, PhD, is responsible for the manuscript entitled “Investigation of metal toxicity on microalgae *Phaeodactylum tricornutum*, hipersaline zooplankter *Artemia salina* and jellyfish *Aurelia aurita*”. On behalf of the rest of the coauthors, with this document, I warrantee and sign that the datasets generated and used during the current study are available from the corresponding author upon reasonable request.

## References

[1] Li Z.H., He K., Liu C., Li P., Zlabek V. (2016). Aquatic Environmental Health and Toxicology. Biomed Res. Int..

[2] Abowey J.F.N., Sikoki F.D. (2007). Water Pollution Management and Control.

[3] Ekubo A.T., Abowi J.F.N. (2011). Aspects of Aquatic Pollution in Nigeria. Res. J. Environ. Earth Sci..

[4] Hampel M., Blasco J., Segner H. (2015). Molecular and Cellular Effects of Contamination in Aquatic Ecosystems. Environ. Sci. Pollut. Res..

[5] Baby J., Raj J., Biby E., Sankarganesh P., Jeevitha M., Ajisha S., Rajan S. (2011). Toxic Effect of Heavy Metals on Aquatic Environment. Int. J. Biol. Chem. Sci..

[6] Farombi E., Adelowo O., Ajimoko Y. (2007). Biomarkers of Oxidative Stress and Heavy Metal Levels as Indicators of Environmental Pollution in African Cat Fish (*Clarias gariepinus*) from Nigeria Ogun River. Int. J. Environ. Res. Public Health.

[7] Khayatzadeh J., Abbasi E. (2010). The 1 St International Applied Geological Congress.

[8] Singh J. (2011). Effects of Heavy Metals on Soil, Plants, Human Health and Aquatic Life. Int. J. Res. Chem. Environ..

[9] Alloway B.J. (2013). Heavy Metals in Soils.

[10] Launay M.A., Dittmer U., Steinmetz H. (2016). Organic Micropollutants Discharged by Combined Sewer Overflows—Characterisation of Pollutant Sources and Stormwater-Related Processes. Water Res..

[11] Fifield F.W., Haines P.J. (2000). Environmental Analytical Chemistry.

[12] Loska K., Wiechuła D. (2003). Application of Principal Component Analysis for the Estimation of Source of Heavy Metal Contamination in Surface Sediments from the Rybnik Reservoir. Chemosphere.

[13] Harguinteguy C.A., Cirelli A.F., Pignata M.L. (2014). Heavy Metal Accumulation in Leaves of Aquatic Plant Stuckenia Filiformis and Its Relationship with Sediment and Water in the Suquía River (Argentina). Microchem. J..

[14] Bashir I., Lone F.A., Bhat R.A., Mir S.A., Dar Z.A., Dar S.A. (2020). Concerns and Threats of Contamination on Aquatic Ecosystems. Bioremediation and Biotechnology.

[15] Zhou Q., Zhang J., Fu J., Shi J., Jiang G. (2008). Biomonitoring: An Appealing Tool for Assessment of Metal Pollution in the Aquatic Ecosystem. Anal. Chim. Acta.

[16] Phillips D.J.H. (1977). The Use of Biological Indicator Organisms to Monitor Trace Metal Pollution in Marine and Estuarine Environments—A Review.

[17] Calzoni G.L., Antognoni F., Pari E., Fonti P., Gnes A., Speranza A. (2007). Active Biomonitoring of Heavy Metal Pollution Using Rosa Rugosa Plants. Environ. Pollut..

[18] Grant A., Trompf K., Seung D., Nivison-Smith L., Bowcock H., Kresse H., Morrow P. (2010). Sub-cellular damage by copper in the cnidarian Zoanthus robustus. Comp. Biochem. Physiol. Part C Toxicol. Pharmacol..

[19] Burger J. (2006). Bioindicators: A Review of Their Use in the Environmental Literature 1970–2005. Environ. Bioindic..

[20] Nordberg G.F. (2009). Historical Perspectives on Cadmium Toxicology. Toxicol. Appl. Pharmacol..

[21] Pavlaki M.D., Araújo M.J., Cardoso D.N., Silva A.R.R., Cruz A., Mendo S., Soares A.M.V.M., Calado R., Loureiro S. (2016). Ecotoxicity and Genotoxicity of Cadmium in Different Marine Trophic Levels. Environ. Pollut..

[22] Chandurvelan R., Marsden I.D., Gaw S., Glover C.N. (2013). Waterborne Cadmium Impacts Immunocytotoxic and Cytogenotoxic Endpoints in Green-Lipped Mussel, Perna Canaliculus. Aquat. Toxicol..

[23] Engel D.W., Fowler B.A. (1979). Factors Influencing Cadmium Accumulation and Its Toxicity to Marine Organisms. Environ. Health Perspect..

[24] Hadjispyrou S., Kungolos A., Anagnostopoulos A. (2001). Toxicity, Bioaccumulation, and Interactive Effects of Organotin, Cadmium, and Chromium on Artemia Franciscana. Ecotoxicol. Environ. Saf..

[25] Yuleidis González Pérez L., Patricia L., Gilling A. (2001). Determinación de la Toxicidad Aguda del Dicromato de Potasio en Larvas de Artemia salina.

[26] Lopez J.S., Lee L., Mackey K.R.M. (2019). The Toxicity of Copper to Crocosphaera Watsonii and Other Marine Phytoplankton: A Systematic Review. Front. Mar. Sci..

[27] Nriagu J.O. (1979). Global Inventory of Natural and Anthropogenic Emissions of Trace Metals to the Atmosphere. Nature.

[28] Duce R.A., Liss P.S., Merrill J.T., Atlas E.L., Buat-Menard P., Hicks B.B., Miller J.M., Prospero J.M., Arimoto R., Church T.M. (1991). The Atmospheric Input of Trace Species to the World Ocean. Glob. Biogeochem. Cycles.

[29] Prospero J.M. (1999). Long-Term Measurements of the Transport of African Mineral Dust to the Southeastern United States: Implications for Regional Air Quality. J. Geophys. Res. Atmos..

[30] Maenhaut W., Salma I., Cafmeyer J., Annegarn H.J., Andreae M.O. (1996). Regional Atmospheric Aerosol Composition and Sources in the Eastern Transvaal, South Africa, and Impact of Biomass Burning. J. Geophys. Res..

[31] Casado-Martínez M. (2006). Interlaboratory Assessment of Marine Bioassays to Evaluate the Environmental Quality of Coastal Sediments in Spain. III. Bioassay using embryos sea urchin *Paracentrotus lividus*. Cienc. Mar..

[32] Gambardella C., Costa E., Piazza V., Fabbrocini A., Magi E., Faimali M., Garaventa F. (2015). Effect of Silver Nanoparticles on Marine Organisms Belonging to Different Trophic Levels. Mar. Environ. Res..

[33] Topçu N.E., Martell L.F., Yilmaz I.N., Isinibilir M. (2018). Benthic Hydrozoans as Potential Indicators of Water Masses and Anthropogenic Impact in the Sea of Marmara. Mediterr. Mar. Sci..

[34] Daly M., Brugler M.R., Cartwright P., Collins A.G., Dawson M.N., Fautin D.G., France S.C., Mcfadden C.S., Opresko D.M., Rodriguez E. (2007). The Phylum Cnidaria: A Review of Phylogenetic Patterns and Diversity 300 Years after Linnaeus*. Zootaxa.

[35] Boero F., Bouillon J., Gravili C., Miglietta M., Parsons T., Piraino S. (2008). Gelatinous Plankton: Irregularities Rule the World (Sometimes). Mar. Ecol. Prog. Ser..

[36] Epstein H.E., Templeman M.A., Kingsford M.J. (2016). Fine-Scale Detection of Pollutants by a Benthic Marine Jellyfish. Mar. Pollut. Bull..

[37] Richardson A.J., Bakun A., Hays G.C., Gibbons M.J. (2009). The Jellyfish Joyride: Causes, Consequences and Management Responses to a More Gelatinous Future. Trends Ecol. Evol..

[38] Sweetman A.K., Smith C.R., Dale T., Jones D.O.B. (2014). Rapid Scavenging of Jellyfish Carcasses Reveals the Importance of Gelatinous Material to Deep-Sea Food Webs. Proc. R. Soc. B Biol. Sci..

[39] Hays G.C., Doyle T.K., Houghton J.D.R. (2018). A Paradigm Shift in the Trophic Importance of Jellyfish?. Trends Ecol. Evol..

[40] Sullivan B.K., Garcia J.R., Klein-Macphee G. (1994). Prey Selection by the Scyphomedusan Predator Aurelia Aurita.

[41] Purcell J. (2003). Predation on Zooplankton by Large Jellyfish, Aurelia, Cyanea and Aequorea, in Prince William Sound, Alaska. Mar. Ecol. Prog. Ser..

[42] Colin S.P., Costello J.H., Graham W.M., Higgins J.I. (2005). Omnivory by the Small Cosmopolitan Hydromedusa Aglaura Hemistoma. Limnol. Oceanogr..

[43] Pitt K.A., Clement A.-L., Connolly R.M., Thibault-Botha D. (2008). Predation by Jellyfish on Large and Emergent Zooplankton: Implications for Benthic–Pelagic Coupling. Estuar. Coast. Shelf Sci..

[44] Macali A., Bergami E. (2020). Jellyfish as Innovative Bioindicator for Plastic Pollution. Ecol. Indic..

[45] Costa E., Gambardella C., Piazza V., Vassalli M., Sbrana F., Lavorano S., Garaventa F., Faimali M. (2020). Microplastics Ingestion in the Ephyra Stage of *Aurelia* Sp. triggers acute and behavioral responses. Ecotoxicol. Environ. Saf..

[46] Almeda R., Wambaugh Z., Chai C., Wang Z., Liu Z., Buskey E.J. (2013). Effects of Crude Oil Exposure on Bioaccumulation of Polycyclic Aromatic Hydrocarbons and Survival of Adult and Larval Stages of Gelatinous Zooplankton. PLoS ONE.

[47] Echols B.S., Smith A.J., Gardinali P.R., Rand G.M. (2016). The Use of Ephyrae of a Scyphozoan Jellyfish, Aurelia Aurita, in the Aquatic Toxicological Assessment of Macondo Oils from the Deepwater Horizon Incident. Chemosphere.

[48] Faimali M., Garaventa F., Piazza V., Costa E., Greco G., Mazzola V., Beltrandi M., Bongiovanni E., Lavorano S., Gnone G. (2014). Ephyra Jellyfish as a New Model for Ecotoxicological Bioassays. Mar. Environ. Res..

[49] Lechable M., Jan A., Duchene A., Uveira J., Weissbourd B., Gissat L., Collet S., Gilletta L., Chevalier S., Leclère L. (2020). An Improved Whole Life Cycle Culture Protocol for the Hydrozoan Genetic Model Clytia Hemisphaerica. Biol. Open.

[50] Lucas C.H. (2001). Reproduction and Life History Strategies of the Common Jellyfish, Aurelia Aurita. Hydrobiologia.

[51] van der Veer H.W., Oorthuysen W. (1985). Abundance, Growth and Food Demand of the Scyphomedusa Aurelia Aurita in the Western Wadden Sea. Neth. J. Sea Res..

[52] Carré D., Carré C. (2000). Origin of Germ Cells, Sex Determination, and Sex Inversion in Medusae of the Genus Clytia (Hydrozoa, Leptomedusae): The Influence of Temperature. J. Exp. Zool..

[53] Larsen G.D. (2016). Unraveling the Mysteries of the Medusa. Lab. Anim..

[54] Houliston E., Momose T., Manuel M. (2010). Clytia Hemisphaerica: A Jellyfish Cousin Joins the Laboratory. Trends Genet..

[55] Bae M.-J., Park Y.-S. (2014). Biological Early Warning System Based on the Responses of Aquatic Organisms to Disturbances: A Review. Sci. Total Environ..

[56] Uba B.O. (2019). Effects of Aromatic Hydrocarbons and Marine Sediments from Niger Delta on the Growth of Microalga *Phaeodactylum tricornutum*. Biotechnol. J. Int..

[57] Pastorino P., Broccoli A., Anselmi S., Bagolin E., Prearo M., Barceló D., Renzi M. (2022). The Microalgae *Chaetoceros tenuissimus* Exposed to Contaminants of Emerging Concern: A Potential Alternative to Standardized Species for Marine Quality Assessment. Ecol. Indic..

[58] Piccardo M., Provenza F., Grazioli E., Anselmi S., Terlizzi A., Renzi M. (2021). Impacts of Plastic-Made Packaging on Marine Key Species: Effects Following Water Acidification and Ecological Implications. J. Mar. Sci. Eng..

[59] Clément L., Hurel C., Marmier N. (2013). Toxicity of TiO_2_ Nanoparticles to Cladocerans, Algae, Rotifers and Plants—Effects of Size and Crystalline Structure. Chemosphere.

[60] Si D., Yang L., Yan H., Wang Q. (2009). Bioaccumulation and Transformation of Cadmium by *Phaeodactylum tricornutum*. Sci. China B Chem..

[61] Okamoto O.K., Asano C.S., Aidar E., Colepicolo P. (1996). Effects of Cadmium on Growth and Superoxide Dismutase Activity of the Marine Migroalga *Tetraselmis gracilis*. J. Phycol..

[62] Wang L., Zheng B. (2008). Toxic Effects of Fluoranthene and Copper on Marine Diatom *Phaeodactylum tricornutum*. J. Environ. Sci..

[63] Jung S.M., Bae J.S., Kang S.G., Son J.S., Jeon J.H., Lee H.J., Jeon J.Y., Sidharthan M., Ryu S.H., Shin H.W. (2017). Acute Toxicity of Organic Antifouling Biocides to Phytoplankton Nitzschia Pungens and Zooplankton Artemia Larvae. Mar. Pollut. Bull..

[64] Franklin N.M., Stauber J.L., Apte S.C., Lim R.P. (2002). Effect of Initial Cell Density on the Bioavailability and Toxicity of Copper in Microalgal Bioassays. Environ. Toxicol. Chem..

[65] Rico M., López A., Santana-Casiano J.M., Gonzàlez A.G., Gonzàlez-Dàvila M. (2013). Variability of the Phenolic Profile in the Diatom *Phaeodactylum tricornutum* Growing under Copper and Iron Stress. Limnol. Oceanogr..

[66] Zulkifli S.Z., Aziz F.Z.A., Ajis S.Z.M., Ismail A. (2014). Nauplii of Brine Shrimp (*Artemia salina*) as a Potential Toxicity Testing Organism for Heavy Metals Contamination. From Sources to Solution.

[67] Fichet D., Miramand P. (1998). Vanadium Toxicity to Three Marine Invertebrates Larvae: Crassostrea Gigas, Paracentrotus Lividus and Artemia Salina. Chemosphere.

[68] Laughlin R.B., Ng J., Guard H.E. (1981). Hormesis: A Response to Low Environmental Concentrations of Petroleum Hydrocarbons. Science (1979).

[69] Karbassi A., Bidhendi G.N., Pejman A., Bidhendi M.E. (2010). Environmental Impacts of Desalination on the Ecology of Lake Urmia. J. Great Lakes Res..

[70] Sarabia R., Del R.J., Varo I., Díaz-Mayans J., Torreblanca A. (2002). Comparing the Acute Response to Cadmium Toxicity of Nauplii from Different Populations of Artemia. Environ. Toxicol. Chem..

[71] Kalčíková G., Zagorc-Končan J., Žgajnar Gotvajn A. (2012). *Artemia salina* acute immobilization test: A possible tool for aquatic ecotoxicity assessment. Water Sci. Technol..

[72] Umarani R., Kumaraguru A.K., Nagarani N. (2012). Investigation of Acute Toxicity of Heavy Metals in Artemia Salina Acclimated to Different Salinity. Toxicol. Environ. Chem..

[73] Eduardo Ruiz González L., Vega-Villasante F. (2014). Evaluación de La Toxicidad de Algunos Basidiomycetes Del Estado de Jalisco Sobre *Artemia franciscana*. Tesis Maest. Cienc..

[74] Eisler R. (1971). Cadmium Poisoning in Fundulus Heteroclitus (Pisces: Cyprinodontidae) and Other Marine Organisms. J. Fish. Res. Board. Can..

[75] Madhav M.R., David S.E.M., Kumar R.S.S., Swathy J.S., Bhuvaneshwari M., Mukherjee A., Chandrasekaran N. (2017). Toxicity and Accumulation of Copper Oxide (CuO) Nanoparticles in Different Life Stages of Artemia Salina. Environ. Toxicol. Pharmacol..

[76] Lucas C.H., Horton A.A. (2014). Short-Term Effects of the Heavy Metals, Silver and Copper, on Polyps of the Common Jellyfish, Aurelia Aurita. J. Exp. Mar. Biol. Ecol..

[77] Karntanut W., Pascoe D. (2002). The Toxicity of Copper, Cadmium and Zinc to Four Different Hydra (Cnidaria: Hydrozoa). Chemosphere.

